# A Broken Heart

**Published:** 1875-08

**Authors:** 


### A BROKEN HEART.

It is not all imagination, not all the invention of poets and fiction-writers, but it is a plain fact, that people do die of a broken heart. The doctors call it rupture of the muscular walls of the ventricle. A change of tissue, softening or fatty degeneration pre-disposes to rupture, as does that condition known as enlargement with dilatation, in which the cavities are increased at the expense of the walls. In this case the heart may be unnaturally distended with blood as in violent exercise, and rupture occurs at the moment of contraction. Mental emotions powerfully affect the heart.

Everybody knows this from his own experience. Palpitation from sudden fright is one of the most common examples. Pain in the heart from continued and crushing anxiety is not an unfrequent symptom. Violent emotions have produced rupture of the heart. A case of this kind was lately reported in the London medical journals, and other cases are recorded. It is not likely that all sudden deaths consequent on mental shock are immediately caused by lesion of the cardiac walls. Paralysis, which is the opposite condition of unyielding contraction or cramp, is equally fatal. Mark Antony understood something of the effect of overwhelming mental emotion, and with rhetorical license ascribes Cæsar's death to a broken heart. He might have borne the "three-and-thirty-wounds," but when his dearest friend, the "well-beloved Brutus, stabb'd," — "ingratitude stronger than traitors' arms quite vanquished him,"— "*then burst his noble heart.*"

